# Magnetic resonance imaging parameter optimizations for diagnosis of periprosthetic infection and tumor recurrence in artificial joint replacement patients

**DOI:** 10.1038/srep36995

**Published:** 2016-11-14

**Authors:** Mei-hua Jiang, Chuan He, Jian-min Feng, Zhi-hui Li, Zhe Chen, Fu-hua Yan, Yong Lu

**Affiliations:** 1 Department of Radiology, Ruijin Hospital, Shanghai Jiaotong University School of Medicine, Shanghai, 200025, China; 2 Department of Orthopedic, Ruijin Hospital, Shanghai Jiaotong University School of Medicine, Shanghai, 200025, China

## Abstract

To evaluate the efficacy of magnetic resonance imaging (MRI) parameter optimizations for the diagnosis of periprosthetic infection and tumor recurrence in joint replacement patients. We compared the quality of images for 16 joint replacement patients that were recorded using the optimized MRI parameters with and without view angle tilting (VAT) correction at 1.5 T in coronal fast-spin-echo T2-weighted MRI. The optimized MRI data of 86 patients with pain after hip replacement and 67 patients who received tumor resection and joint replacement for bone cancer were retrospectively analyzed to identify MRI features that were useful for the diagnosis of periprosthetic infection and tumor recurrence. Increasing receiver bandwidth and decreasing slice thickness combined with VAT significantly reduced the area of metal-induced artifacts. Irregular soft tissue mass, soft tissue edema, bone destruction, and fistula were significant features of periprosthetic infection, with sensitivities of 47.4–100% and specificities of 73.1–100.0%, which were confirmed based on surgical and pathological findings. Soft tissue mass was a significant feature of tumor recurrence, with 100% sensitivity, 96.0% specificity, and 97.0% consistency. The optimized VAT MRI method demonstrated a high level of diagnostic accuracy for the detection of periprosthetic infection and tumor recurrence in joint replacement patients.

Magnetic resonance imaging (MRI) has been widely used in postoperative follow-up for hip replacement patients because of its higher resolution for bone and soft tissue. However, the metal components of the prosthesis distort the static magnetic field created during MRI. These changes in the magnetic field can corrupt the slice selection and frequency encoding gradients, which causes through-plane and in-plane artifacts in the imaging data. Such imaging artifacts often confound the diagnosis of postoperative complications, such as periprosthetic infection and a loose fitting implant^1^,^2^,^3^.

A previous study showed that MRI data obtained using a syngo WARP optimized sequence, which used higher bandwidth, thinner slicing, and view angle tilting (VAT), had fewer metal-induced artifacts and better imaging quality, compared with a routinely used MRI sequence^4^. Generally speaking, the use of higher receiver bandwidth, which increases the magnetic strength of the frequency encoding gradient, can mitigate the influence of metal of the susceptibility gradient^5^,^6^. Using VAT, a compensated slice-selection gradient can be created along the slice-selection axis during signal read-out, which results in a shearing effect in the imaging slice that is equivalent to a tilting view angle. Therefore, certain in-plane geometric distortions, such as those caused by metal prostheses, can be corrected using VAT.

The ability to correct such metal-induced artifacts has improved the postoperative assessment of artificial joint replacements. However, the quality of MRI data obtained using metal artifact correction methods remains suboptimal for the diagnosis of many postoperative complications, including the detection of infection and tumor recurrence. Using paraffin models with a metal alloy hip prostheses, we optimized the receiver bandwidth and slice thickness parameters of a VAT MRI technique at 1.5 T in an effort to improve the diagnostic accuracy of MRI for detecting periprosthetic infection and tumor recurrence, and we evaluated the sensitivity and specificity of the optimized VAT MRI method based on the surgical and pathological findings for joint replacement patients.

## Patients and Methods

### Hip prosthesis models

Titanium alloy and cobalt chromium molybdenum alloy hip prostheses were fixed in paraffin, and scanned using fast-spin-echo (FSE) T2-weighted MRI scanning (T2WI) at 1.5 T. While keeping other parameters constant, the artifact areas were measured using different receiver bandwidths and slice thicknesses using an ADW4.4 workstation, as shown in Table 1. The artifact area measurements were recorded using periprosthetic adjacent signal loss for pile-up artifacts, as described previously^7^.

### Evaluation of metal-induced artifacts

Imaging data for 16 joint replacement patients were recorded using simple MRI parameter optimizations (higher receiver bandwidth and thinner slice) alone and in combination with VAT for coronal FSE T2WI MRI scanning at 1.5 T. Imaging quality scores of 1 to 5 were independently assigned by two radiology residents as follows: 5, high quality image with no artifacts; 4, contained minimally detectable artifacts; 3, contained significant artifacts that did not affect diagnostic quality; 2 contained artifacts that affected diagnostic quality; or 1 contained artifacts that prevented diagnosis. A score of ≥3 was considered sufficient for diagnosis. The nonparametric rank sum test was used to compare the image quality scores obtained using the different optimization methods.

### Patients

Our study was approved by the Ethics Committee of Ruijin Hospital and Shanghai Jiao Tong University. Signed informed consent was obtained from all of the patients before their participation in our study. Our study was performed in accordance with the Declaration of Helsinki regarding the ethical principles for medical research involving human subjects. Initially, the MRI data from 16 joint replacement patients were used to compare the image quality scores of datasets obtained using the optimized MRI settings in combination with and without the VAT technique.

We then conducted a retrospective study, in which we analyzed MRI data obtained using the optimized VAT method for 86 patients (34 men and 52 women) with a mean age of 67 years (range: 30–89 years) with pain after hip replacement and 67 patients (41 men and 26 women) with a mean age of 26 years (range: 9–64 years) after tumor resection and joint replacement. All of the patients were treated at Ruijin Hospital from January 2014 to August 2016, and received T1, T2, and short-time inversion recovery (STIR) MRI in a Magnetom Aera 1.5 T Superconducting MRI scanner (Siemens Healthcare, Erlangen, Germany).

### Retrospective analysis of MRI data

For patients who underwent hip replacement only (n = 86), scans were performed 1 week before revision surgery. Patients who underwent bone tumor resection with joint replacement received plain MRI scanning (n = 65) or enhanced scanning (n = 2) at 3 months following surgery. The MRI features, including periprosthetic irregular soft tissue mass, soft tissue edema, periprosthetic joint effusion, bone edema, bone loss, and fistula formation were compared to the surgical and pathological results using a two-sample chi-squared test. Each type of MRI feature was evaluated for diagnosing postoperative tumor recurrence and infections based on comparison with surgical and pathological findings, and the occurrence of the selected MRI features were compared between the recurrence and non-recurrence cases as well as the infection and non-infection cases separately, using a chi-squared analysis. The consistency of the MRI features for the diagnosis of tumor recurrence and infections were analyzed using a Kappa consistency analysis.

Periprosthetic joint infections were diagnosed when bacterial cultures of the joint fluid and joint capsule biopsies were positive or when >5 neutrophils were detected in periprosthetic tissues at high magnification^8^. The diagnostic criteria of tumor recurrence were MRI imaging and pathology confirmations of resected tissues or biopsies. All recurrence patients received chemotherapy.

## Results

### Optimization and assessment of the MRI procedure

**Figure 1:**
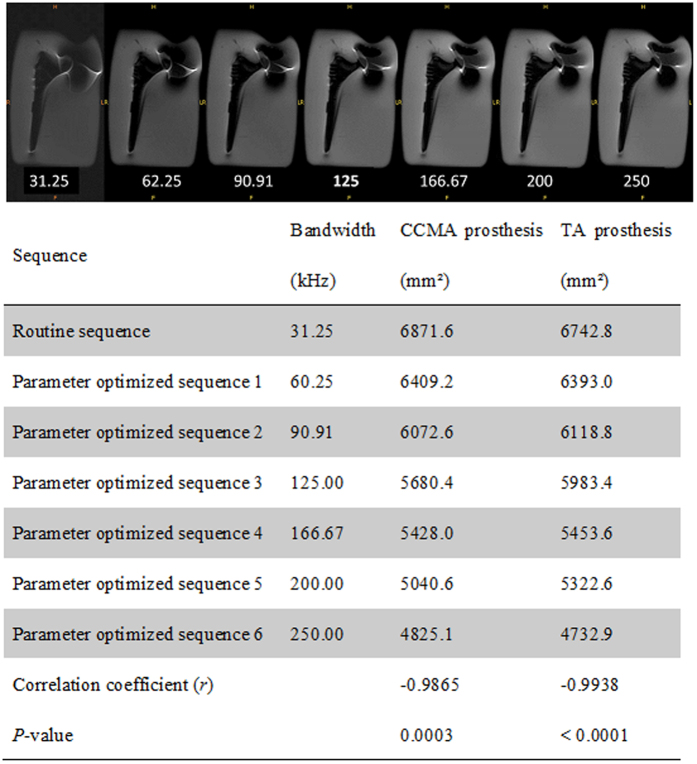
Correlation of bandwidth with the artifact areas for the cobalt chromium molybdenum alloy (CCMA) and titanium alloy (TA) prostheses.

**Figure 2:**
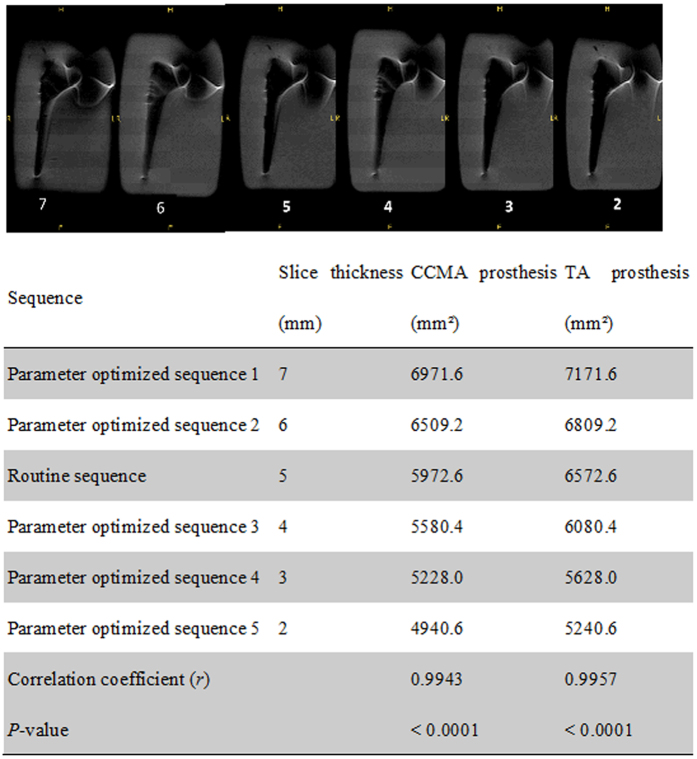
Correlation of slice thickness with the artifact areas for the cobalt chromium molybdenum alloy (CCMA) and titanium alloy (TA) prostheses.

**Figure 3:**
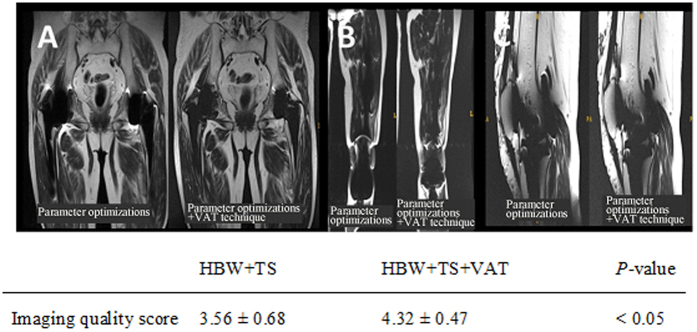
Representative images of patients scanned using the optimized MRI parameters (high bandwidth [HBW] and thin slice [TS]) with or without VAT. **(A)** MRI of hip joint prosthesis showing (Left) imaging with simple parameter optimizations and (Right) imaging with parameter optimizations and VAT. **(B)** MRI of joint prosthesis showing (Left) imaging with simple parameter optimizations and (Right) imaging with parameter optimizations and VAT. **(C)** Knee joint prosthesis MRI showing (Left) imaging with simple parameter optimizations and (Right) imaging with parameter optimizations and VAT.

Using the paraffin-embedded titanium alloy and cobalt chromium molybdenum alloy hip prosthesis models, increasing bandwidth from 31.25 kHz to 60.25, 90.91, 125, 166.67, 200, or 250 kHz, while holding other parameters constant, reduced artifacts and improved imaging quality, with negative correlations observed between bandwidth and artifact occurrence for the titanium alloy (*r* = −0.9938, *P* = < 0.0001) and cobalt chromium molybdenum alloy (*r* = −0.9865, *P* = 0.0003) hip prosthesis models (Fig. 1). Reduced artifacts and improved imaging quality were also achieved by decreasing slice thickness (from 7 mm to 6, 5, 4, 3, or 2 mm), with a positive correlation observed between slice thickness and artifact occurrence for the titanium alloy (*r* = 0.9957, *P* = < 0.0001) and cobalt chromium molybdenum alloy (*r* = 0.9943, *P* = <0.0001) hip prosthesis models (Fig. 2). The optimized MRI parameters combined with the VAT technique produced fewer artifacts and higher image quality than those achieved using MRI with the optimized parameters and no VAT, with mean imaging scores of 4.32 ± 0.47 and 3.56 ± 0.68, respectively (*P* < 0.05; Fig. 3).

### VAT MRI for postoperative assessment of hip replacement complications

The optimized VAT MRI method was used in T1, T2, or STIR sequence for the assessment of patients reporting pain following hip replacement. The main MRI features of periprosthetic infection observed were irregular periprosthetic soft tissue mass, bone edema, hydrarthrosis, bone destruction, and sinus tract, and extensive soft tissue edema, which were present in all of the 19 cases of periprosthetic infection. The occurrence of irregular periprosthetic soft tissue mass, extensive soft tissue edema, bone destruction, and sinus tract among the patients with periprosthetic infection were significantly greater than those in patients with no infection (*P* < 0.001), indicating their value for the diagnosis of periprosthetic infection of the hip (Table 2).

### VAT MRI for postoperative assessment of joint replacement due to bone cancer

The optimized VAT MRI method was used in T1, T2, or STIR sequence to detect tumor recurrence in patients who had undergone joint replacement due to bone cancer. Fifty of the 67 bone cancer patients were free of tumor recurrence, among whom 47 patients were deemed healthy (no periprosthetic soft tissue mass), 2 were diagnosed with periprosthetic infection (confirmed by surgery), and one was diagnosed as having a dislocated patella (confirmed by clinical examination). The 17 remaining patients were found to have suffered tumor recurrence (Table 3).

**Figure 4:**
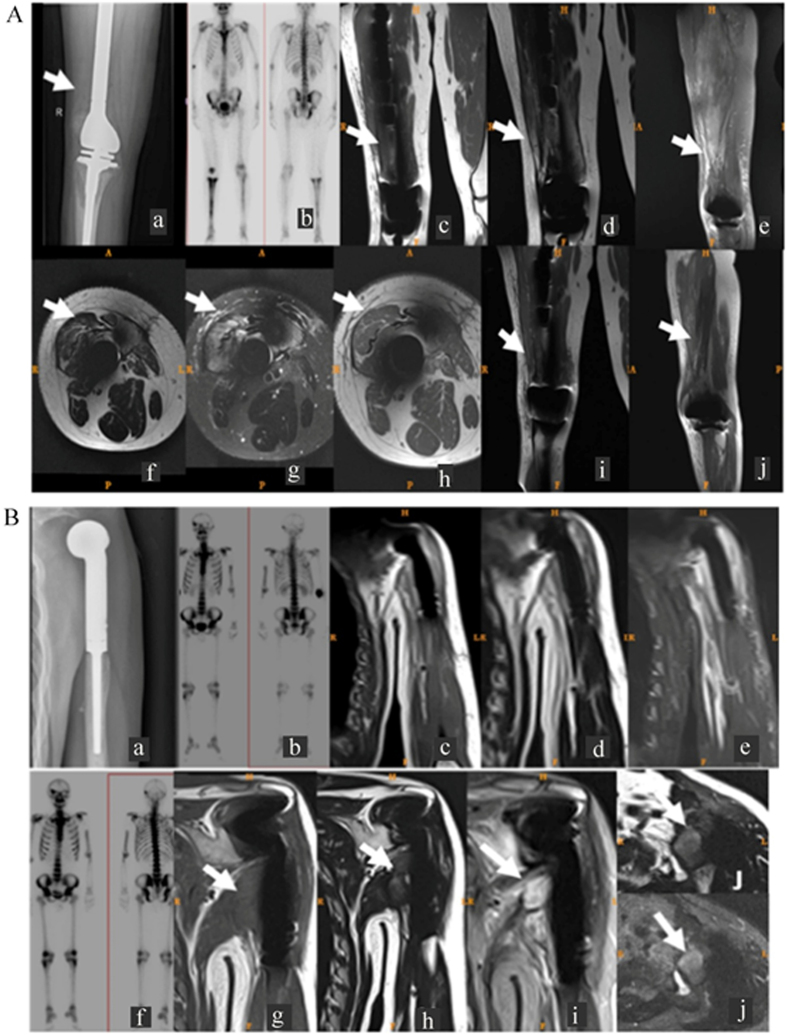
MRI features of joint replacement patients with or without tumor recurrence. **(A)** Imaging results for a 19-year-old male patient with right femoral osteosarcoma who received femoral segment resection and joint replacement, with no sign of tumor recurrence at 3 months postsurgery. (Aa) Anteroposterior X-ray showed the affected knee joint prosthesis was positioned well. The surrounding soft tissue was swollen, but no abnormal soft tissue mass was present. (Ab) Whole body bone scan showed radioactivity uptake enrichment at the affected knee joint and tibiofibula. Follow-up MRI included (Ac) coronal T1WI, (Ad) coronal T2WI, (Ae) sagittal STIR, (Af) transverse T2WI, and (Ag) STIR plain images and (Ah) transverse, (Ai) coronal, and (Aj) sagittal T1WI enhanced images. **(B)** Imaging results for a 33-year-old male patient following left humeral proximal osteosarcoma tumor segment resection and joint replacement. (Ba) Anteroposterior X-ray of affected shoulder joint showing well-positioned prosthesis and normal surrounding soft tissue. (Bb) Whole body bone scan showing radioactivity uptake enrichment on the distal affected humeral bone. Follow-up MRI at 3 months postsurgery included (Bc–Be) coronal T1WI, T2WI, and STIR scans, which showed fuzzy muscle structures around the prosthesis. Higher signal on the T2WI and STIR images revealed no obvious abnormal soft tissue mass around implants. (Bf) Whole body bone scan showed radioactivity uptake enrichment at the distal affected humeral bone, which was similar with the previous image. Follow-up MRI at 10 months postsurgery included (Bg–Bi) coronal T1WI, T2WI, and STIR images and transverse (Bj, upper) T2WI and (Bj, lower) STIR images, which showed increased nodular soft tissue signals in the inferior left shoulder joint that was a relatively low signal in the T1WI images, a slightly higher signal in the T2WI images, and a mixed high and low signal in the STIR images (arrow). Periprosthetic bone edema was also observed.

Figure 4A shows the MRI of a well-positioned prosthesis after right femoral osteosarcoma segment resection with joint replacement. There was disordered periprosthetic muscle structure, fuzzy muscular clearance, and increased signal intensity on T2 and STIR for some muscles with no improved clarity in the enhanced images. Right thigh subcutaneous fat appeared as an abnormal patchy signal, which was low in T1, slightly higher in T2, and extremely high in STIR images. The images in Fig. 4B show local recurrence of osteosarcoma at 10 months postsurgery in a 33-year-old male patient who underwent surgical resection and joint replacement for an osteosarcoma tumor of the proximal left humerus.

Tumor absence was manifested primarily as reduced periprosthetic soft tissue swelling and effusion following surgery (Fig. 4A), whereas tumor recurrence was manifested primarily as nodular or crumb abnormal signal of soft tissue (Fig. 4B).

Periprosthetic soft tissue mass and bone destruction were significantly more common in patients suffering recurrence than in patients who were cancer free (*P* < 0.05) (Table 4) and periprosthetic soft tissue mass demonstrated a high level of consistency for diagnosing tumor recurrence (Table 5).

### Diagnostic accuracies of the optimized VAT MRI procedure

As shown in Table 6, the sensitivity of the optimized VAT MRI method for diagnosing periprosthetic infection in hip replacement patients was 52.6% for soft tissue mass, 100% for soft tissue edema, 47.4% for bone destruction, and 47.4% for fistula. The specificity was 89.6% for soft tissue mass, 73.1% for soft tissue edema, 92.5% for bone destruction, and 100% for fistula. The detection of fistula demonstrated the highest diagnostic consistency (88.4%), which was followed by bone destruction (82.6%), soft tissue mass (81.4%) and soft tissue edema (79.1%). The sensitivity, specificity, and consistency of soft tissue mass for diagnosing tumor recurrence in joint replacement patients were 100%, 96.0%, and 97.0%, respectively.

## Discussion

Although MRI has been widely used for tumor detection and the visualization of adjacent soft tissue in bone cancer patients^9^, X-ray or computed tomography are often used for patients receiving joint replacement due to the occurrence of metal-induced MRI artifacts. Techniques have been developed for reducing metal-induced MRI artifacts. However, few studies have investigated the diagnostic value of MRI features in images obtained using metal-induced artifact reduction techniques for detecting tumor recurrence in bone cancer patients receiving joint replacement.

In our current study, we investigated the effect of parameter optimization (high receiver bandwidth or thin slice) on the reduction of metal-induced artifacts in VAT MRI for patients with joint replacement, and assessed the diagnostic value of the optimized VAT MRI technique for detecting tumor recurrence in bone cancer patients following concommitant joint replacement. Optimization significantly improved imaging quality and decreased artifact area, and certain MRI features, especially soft tissue mass, demonstrated a high level of consistency for the diagnosis of tumor recurrence. Tumor recurrence was characterized by nodular or crumby soft tissue signal around the prosthesis. In the 67 patients examined in the evaluation of the usefulness of the MRI features for diagnosing tumor recurrence, the soft tissue mass feature was observed in all of the 17 patients with surgically confirmed tumor recurrence, demonstrating high levels of sensitivity (100%) and specificity (96.0%). To our knowledge, this is the first report of the diagnostic accuracy of an MRI feature for detecting tumor recurrence.

The use of MRI to confirm the findings of radiographs or clinical examination has been shown to be effective for the detection of local tumor recurrence^10^. However, no single MRI feature has demonstrated a high level of diagnostic value individually, and a comprehensive comparison of the diagnostic accuracy of a single MRI feature and that of clinical examination, laboratory testing, or X-ray radiography has not been reported. Nuclear medicine is highly effective for the diagnosis of tumor recurrence, but its clinical use is often limited due to higher cost. Our optimized VAT MRI technique requires no additional devices, and does not increase scanning time, providing a cost-effective method for the diagnosis of tumor recurrence. Although the patient sample used to evaluate the accuracy of our optimized VAT MRI method was relatively small, the high levels of sensitivity (100%) and specificity (96.0%) demonstrated in our analysis warrant future large-scale investigations of the diagnostic value of the optimized VAT MRI method for detecting tumor recurrence.

We also evaluated the use of the optimized VAT MRI technique for detecting periprosthetic infection following joint replacement, which is a relatively uncommon complication. The diagnosis of periprosthetic infection often requires multiple diagnostic strategies, and an optimal diagnostic method has not been determined. In a previous study, White *et al.* modified standard MRI sequences, and demonstrated consistent diagnostic accuracy for detecting complications following total hip replacement^3^. Based on their findings, we hypothesized that the optimization of MRI parameters combined with VAT would allow the visualization of structural aspects of periprosthetic infection, such as bone edema, bone destruction, joint effusion, soft tissue edema, irregular soft tissue mass formation, and fistula formation. Our evaluation of the diagnostic value of these MRI features, showed that the distribution of soft tissue edema, bone destruction, irregular soft tissue mass formation, and fistula formation was significantly different between patients with postoperative periprosthetic infection and those without infection. These findings indicate that the differential diagnosis of postoperative periprosthetic infection would likely be benefited by the application of these indices. However, our optimized VAT MRI method did not completely eliminate metal-induced artifacts, and some blurring remained due to the VAT technique. Therefore, the diagnosis of postoperative periprosthetic infection should also consider medical history, clinical examination, and laboratory findings.

Artifacts can generally be reduced by increasing the bandwidth or decreasing the slice thickness, both of which increase the amplitude of the frequency encoding gradient^11^. This relationship was represented in our data by the artifact area positively and negatively correlating with receiver bandwidth and slice thickness, respectively. In a previous study, Toms *et al.* suggested that over 90% of artifacts can be reduced by using matrices of 256 × 256 or greater and a receiver bandwidth of greater than 400 Hz/pixel^12^. This technique lowers the signal-to-noise ratio, which was reflected in our data by the lower imaging scores obtained using larger bandwidths. However, based on our clinical experiences, these reduced imaging scores would not preclude a reliable diagnosis, despite the reduction of the signal to noise ratio.

Multiple studies have demonstrated the advantage of VAT for reducing metal-induced artifacts. Muller *et al.* compared the effects of T1 high bandwidth, STIR high bandwidth, T1 VAT, and T1 VAT combined with slice encoding metal artifact correction (SEMAC) for reducing imaging artifacts, and found that the artifacts area for T1 VAT was smaller than that for TI VAT + SEMAC and T1 high bandwidth^13^. Although VAT can cause image blurring as a result of geometrical tilting of the image pixels^4^, this can be minimized by using shorter high bandwidth readouts^14^. Kolind *et al.* developed a metal artifact reduction sequence consisting of a VAT-SE sequence with high radio frequency and readout bandwidth, which produced enhanced images near metal objects^15^. Therefore, our optimization of bandwidth and slice thickness in combination with VAT for reducing metal-induced artifacts represents a logical extension of the findings of these previous studies, and highlights the clinical applicability of this MRI technique. Limitations of our present study was the retrospective design and the relative small sample size.

In summary, we combined an optimization of receiver bandwidth and slice thickness with VAT MRI to reduce metal-induced artifacts for the postoperative assessment of joint replacement patients. We found that certain MRI features, including periprosthetic irregular soft tissue mass, soft tissue edema, bone destruction, and fistula formation, displayed high levels of sensitivity, specificity, and consistency for the diagnosis of periprosthetic infection in hip replacement patients. Our results also showed that periprosthetic soft tissue mass demonstrated a high level of diagnostic accuracy for the detection of tumor recurrence in osteosarcoma patients following joint replacement. Future clinical studies with larger patient populations are warranted to confirm our findings.

## Additional Information

**How to cite this article**: Jiang, M.-h. *et al.* Magnetic resonance imaging parameter optimizations for diagnosis of periprosthetic infection and tumor recurrence in artificial joint replacement patients. *Sci. Rep.* **6**, 36995; doi: 10.1038/srep36995 (2016).

**Publisher’s note:** Springer Nature remains neutral with regard to jurisdictional claims in published maps and institutional affiliations.

## Figures and Tables

**Table 1:** Optimization sequence of MRI parameters.

Sequence	Bandwidth (kHz)	Slice (mm)	Scanning time (min)
Routine sequence	31.25	5	1:31
Parameter optimized sequence 1	60.25	7	1:31
Parameter optimized sequence 2	90.91	6	1:31
Parameter optimized sequence 3	125	4	1:31
Parameter optimized sequence 4	166.67	3	1:31
Parameter optimized sequence 5	200	2	1:31
Parameter optimized sequence 6	250	—	1:31

**Table 2:** Number and percentage of patients with (n = 19) or without (n = 67) periprosthetic infection after hip replacement who exhibited the various MRI features.

Periprosthetic feature	Infection	No infection	χ^2^	*P*-value
Irregular soft tissue mass	10 (52.6%)	7 (10.4%)	16.608	0.000
Soft tissue edema	19 (100%)	18 (26.9%)	32.298	0.000
Hydrarthrosis	8 (42.1%)	16 (23.9%)	2.444	0.118
Bone Edema	8 (42.1%)	15 (22.4%)	2.937	0.087
Bone destruction	9 (47.4%)	5 (7.5%)	17.296	0.000
Sinus tract	9 (47.4%)	0 (0%)	35.446	0.000

**Table 3:** The tumor locations in patient with (n = 17) or without (n = 50) recurrence after joint replacement.

	Patients with tumor recurrence (n = 17)	Patients without tumor recurrence (n = 50)	*p*-Value
Tumor location			
Proximal femur	7 (41.2%)	4 (8.0%)	0.001
Proximal humerus	5 (29.4%)	4 (8.0%)	0.025
Distal femur	5 (29.4%)	31 (62.0%)	0.020
Proximal tibia	0 (0)	11 (22.0%)	0.034
Pathological diagnosis			
Chondrosarcoma	2 (11.8%)	1 (2.0%)	0.093
Osteosarcoma	14 (82.3%)	44 (88.0%)	0.555
PNET/Ewing	1 (5.9%)	1 (2.0%)	0.416
Bony giant-cell tumor	0 (0)	1 (2.0%)	0.057
Spindle cell sarcoma	0 (0)	2 (4.0%)	0.402
Malignant fibrous histiocytoma	0 (0)	1 (2.0%)	0.557

**Table 4:** Number and percentage of patients with (n = 17) or without (n = 50) tumor recurrence after joint replacement who exhibited the various MRI features.

Periprosthetic feature	No recurrence	Recurrence	χ^2^	*P*-value
Soft tissue mass	2 (4.0%)	17 (100%)	57.549	0.000
Soft tissue edema	40 (80.0%)	11 (64.7%)	1.632	0.201
Effusion	43 (86.0%)	2 (11.8%)	31.701	0.000
Bone edema	11 (22.0%)	3 (17.6%)	0.143	0.703
Bone destruction	1 (1.5%)	5 (29.4%)	11.692	0.001

**Table 5:** Consistency of MRI features for diagnosing tumor recurrence.

Periprosthetic feature	κ-value	*P*-value	Consistency
Soft tissue mass	0.924	0.000	Significant and satisfactory
Soft tissue edema	−0.092	0.201	Without significance
Effusion	−0.481	0.000	Without significance
Bone edema	0.145	0.499	Significant and unsatisfactory
Bone destruction	0.342	0.001	Significant and unsatisfactory

**Table 6:** Sensitivity, specificity, and consistency of optimized VAT MRI for diagnosing periprosthetic infection and tumor recurrence in joint replacement patients.

	Hip replacement (n = 86)				Joint replacement for cancer (n = 67)
Diagnostic variable	Soft tissue mass	Soft tissue edema	Bone destruction	Fistula	Soft tissue mass
Sensitivity	52.6%	100.0%	47.4%	47.4%	100.0%
Specificity	89.6%	73.1%	92.5%	100.0%	96.0%
Consistency	81.4%	79.1%	82.6%	88.4%	97.0%
